# Effects of three regeneration methods on the growth and bacterial community diversity of *Populus × euramericana*

**DOI:** 10.1371/journal.pone.0273306

**Published:** 2022-08-26

**Authors:** Yanyan Fu, Changjun Ding, Jianmin Fan, Yongtan Li, Lizhu Yao, Minsheng Yang, Xiaohua Su, Jinmao Wang

**Affiliations:** 1 Forest Department, Forestry College, Hebei Agricultural University, Baoding, China; 2 State Key Laboratory of Tree Genetics and Breeding, Research Institute of Forestry, Chinese Academy of Forestry, Beijing, China; 3 Key Laboratory of Tree Breeding and Cultivation of State Forestry Administration, Research Institute of Forestry, Chinese Academy of Forestry, Beijing, China; 4 Hebei Key Laboratory for Tree Genetic Resources and Forest Protection, Baoding, China; Shandong University, CHINA

## Abstract

To study the effects of different regeneration methods on the growth and bacterial community diversity of *Populus × euramericana cv*. *‘74/76’* (poplar 107), we investigated the growth of poplar 107 trees under three regeneration methods in 2017 and 2020, and sequenced the 16S rDNA V5–V7 regions in stem endophytic, root endophytic, and rhizosphere soil bacteria present in samples from the three regeneration methods using the Illumina high-throughput sequencing platform. The growth analysis showed that stump grafting regeneration (ST) and stump sprouting regeneration (SP) presented similar tree height and diameter at breast height (DBH), which were significantly lower by planted seedling regeneration (CK). The high-throughput sequencing results showed that the rhizosphere soil bacteria appeared to be significantly more diverse and rich than the root and stem endophytic bacteria. Cluster analysis showed that the similarity of bacterial community structure among the rhizosphere soil, root, and stem was small. Thus, the three sample types showed significant differences in bacteria. While comparing the two years, 2020 was significantly more diverse and rich than 2017. With the increase in stand age, the abundance of Proteobacteria increased and the abundance of Acidobacteria decreased. Among the three regeneration methods, ST significantly increased the diversity of stem endophytic bacteria. *Chthoniobacter* was enriched in SP, which promoted the decomposition of organic matter, and more plant growth promoting rhizobacteria (PGPR) were accumulated in the rhizosphere of SP and ST. The composition of the bacterial community was similar in the three regeneration methods, but the community composition was different. Regeneration and transformation of poplar plantations can be better carried out by stump grafting and stump sprouting.

## 1. Introduction

*Populus*, which is one of the three fast-growing tree species used for the development of plantations, belongs to the family Salicaceae, and has the characteristics of wide distribution, strong adaptability, and many varieties [1]. Poplars play an important role in ecological construction; however, due to natural disasters, degeneration of poplar varieties, and delayed regeneration, low-efficiency poplar forests are widespread [2]. Therefore, it is important to study effective regeneration. At present, the main regeneration methods used for poplar are planted seedling (stem) regeneration, stump sprouting regeneration, and stump grafting regeneration. Planted seedling regeneration is a conventional afforestation method for fast-growing and high-yielding forests in China. It requires the removal of stumps, leveling of the land, and excavation of holes for planting, which may damage the ground surface and results in high afforestation costs [3]. Stump sprouting regeneration is a self-regeneration method in which sprout branches are generated using the stumps and underground roots remaining after the felling of the aboveground parts and the main branches are retained and cultivated after budding and management [4]. This type of regeneration, which retains the original poplar root system, is simple and fast to implement, results in rapid growth, and has low cost. The stump grafting regeneration method uses the stump remaining after the tree is felled as the rootstock and a new poplar scion is used for grafting and regeneration [5]. This technology has been used widely in the regeneration and transformation of poplar forests because of its low investment requirement, simple execution, and high survival rate. Stump grafting and stump sprouting regeneration methods of retaining stumps may have adverse effects on the growth of poplar trees by root system aging and death over time. In addition, stump sprouting seedlings continue to grow and develop on the stumps, which may inherit the physiological characteristics and microbial flora of the stumps, and appear the phenomenon of premature aging. These different regeneration methods differ in whether stumps are retained and used. Therefore, we compared the differences in growth and microbial diversity between the three regeneration methods to determine the beneficial or detrimental effects of stump grafting regeneration and stump sprouting regeneration on the poplar itself and afforested land during the rotation period. The accumulated research remains very limited. Wu et al. [2] investigated the growth indices of 9-year-old poplar 107 planted seedlings and stump-grafted seedlings and found that the growth indices of the stump-grafted stand were significantly higher than the indices of the planted-seedling stand in the same year. Geng et al. [6] compared the growth of a *Robinia pseudoacacia* coptic forest and a first-generation seedling forest and found that the coptic forest had higher tree height than the seedling forest in the first 22 years but had lower height than the seedling forest thereafter. Kammesheidt [7] studied the restoration of tropical moist forest in Paraguay and found that sprouting regeneration increased community biodiversity. Lv et al. [8] studied the diversity of rhizosphere bacteria of *Cerasus sachalinensis* Kom. with grafting and found that grafting promoted the bacteria.

Endophytic bacteria colonize living plants and can maintain a harmonious coexistence with healthy plants [9]. Roots are the organ directly connecting plants with soil, and specific niche changes in microbial communities occur at the interface between roots and soil [10]. As the region of interaction between plants, soil, and microorganisms [11], the rhizosphere is involved in material circulation, energy flow, and information transfer among them [12]. In addition to absorbing water and nutrients, roots are surrounded by complex microbial communities [13], which are directly or indirectly related to the plant achieving mutualism [14, 15]. Many studies have shown that beneficial microorganisms in the rhizosphere make outstanding contributions to coping with abiotic stress and improve the ability of plants to survive in the extreme environments caused by various types of abiotic stress, such as drought stress, salt stress, and nutrient deficiency [16]. At the same time, plants secrete various compounds into the environment, including root exudates, which are closely related to the interactions of the rhizosphere environment [17]. Therefore, research on the diversity of rhizosphere soil microorganisms and endophytic bacteria can help reveal the adaptability of plants to the environment and the relationships between plants and microorganisms. At present, poplar 107 is the main poplar species used as a short-cycle industrial timber forest in North China. Most of them grow best and have the fastest speed in 3–4 years, while the rotation period is generally every 5–7 years. Therefore, we selected the 4-year-old with the most vigorous growth and the 7-year-old that is about to be harvested, compared the effects of three regeneration methods on the growth of poplar 107. We also compared the diversity of the stem, root endophytic, and rhizosphere bacterial communities under the different regeneration methods to provide a reference for the regeneration of poplar and the management of poplar afforestation.

## 2. Materials and methods

### 2.1 Overview of study area and construction of experimental forest

The experimental forest was located in the nursery farm of the village of Maojiaying, Bachigang Town, Luannan County, Tangshan City, Hebei Province (39°12’–39°39’ N, 118°12’–118°53’ E). The study area is within a warm temperate, semi-humid monsoon, continental climate zone having the characteristics of dry winter, wet summer, concentrated precipitation, significant monsoon, and four distinct seasons. The soil texture is sandy. The experimental forest was reconstructed on an existing forest of 10-year-old poplar 107. Because about one-third of the original poplar 107 forest was short of plants or randomly distributed, the stumps produced from cutting in the spring of 2014 were grafted with poplar 107 for regeneration and pits were also dug to plant one-year-old poplar 107 seedlings. Because the survival rate of grafting was about 70% in 2014, the main sprouts of the stumps that were grafted unsurvived were retained, any excess sprouts were removed. Finally, an experimental forest including three regeneration methods, namely, planted seedling regeneration (CK), stump grafting regeneration (ST), and stump sprouting regeneration (SP), was established. In the year of ST and SP regeneration, when the new seedlings growed to a height of 30-50cm, the base of the seedlings was sealed 2–3 times, and the height of the sealed soil was 25-35cm. A completely random block design with four replicates and 30 plants in each plot was adopted. To promote the rapid growth of poplar 107, the experimental forest had a row spacing of 2 m × 5 m, with a tree age of 4 a in 2017 and 7 a in 2020. During the dry and water-deficient season, water was properly replenished, and the litter produced every year was not removed from the experimental forest, but returned directly to the soil.

### 2.2 Sample collection and processing

At the end of each growing season, the height and diameter at breast height (DBH) of the experimental forest were investigated. Ten trees each of CK, SP, and ST were sampled randomly in four blocks each year.

Microbe samples were collected in August of each year. Poplar 107 trees from the three regeneration methods were selected randomly from four blocks, each with nine trees. Stem endophytic samples (E) were collected at the 1.3-m DBH of the target plant. A growth cone sterilized with alcohol was used to drill wood strips of CK, SP, and ST perpendicular to the trunk, and sample from each of three trees was placed into a zip-seal bag as a mixed sample. As three repetitions were used, there were three mixed samples for each regeneration method, for a total of nine stem samples for the three regeneration methods. The root endophytic samples (R) and rhizosphere soil samples (S) were obtained from the same plants as the stem samples. The specific collection methods were as follows: In the four directions, southeast and northwest of the target tree, 0.5m away from the trunk, fine roots in the soil 15-25cm from the surface were collected. After shaking the roots and removing the loose soil from them, the residual soil was collected from the roots with a sterile brush and used as the rhizosphere soil. The brushed roots from the rhizosphere soil were used as the root samples. These were washed five times with sterile water, placed in 15 ml of PBS (phosphate buffer saline) solution, treated with ultrasound at 50–60 Hz for 30 s, and then sterile water-absorbing paper was used to absorb the water remaining on the root surfaces. Finally, nine root and nine rhizosphere soil samples of the three regeneration methods were obtained. In total, 27 samples were collected in both years and stored in the laboratory’s −80°C freezer until high-throughput sequencing could be performed. The sample numbers are shown in Table 1.

**Table 1:** Sample numbers of all samples.

Regeneration methods	Stem endophytic (E)	Root endophytic (R)	Rhizosphere Soil (S)
ST	ST-1E	ST-1R	ST-1S
	ST-2E	ST-2R	ST-2S
	ST-3E	ST-3R	ST-3S
SP	SP-1E	SP-1R	SP-1S
	SP-2E	SP-2R	SP-2S
	SP-3E	SP-3R	SP-3S
CK	CK-1E	CK-1R	CK-1S
	CK-2E	CK-2R	CK-2S
	CK-3E	CK-3R	CK-3S

Note: ST, stump-grafting regeneration; SP, stump-sprouting regeneration; CK, planted seedling regeneration

### 2.3 DNA extraction and Illumina sequencing

Samples with a mass of 3 g were weighed from each mixed sample for DNA extraction and sequencing. Guangzhou Gene Denovo Biotechnology Co., Ltd. (China), was entrusted to implement bacterial-specific primers 799F (5’-AACMGGATTAGA TACCCKG-3’) and 1193R (5’-ACGTCATCCCCACCTTCC-3’) with barcodes for polymerase chain reaction (PCR) amplification of the bacterial 16S rDNA V5–V7 regions, with sequencing performed on the Illumina PE250 platform (Illumina, CA, USA). To ensure statistical reliability and biological validity of the subsequent analyses of the raw data obtained by sequencing, strict quality control was carried out through multiple data-processing processes such as reads utilization, tags splicing, and operational taxonomic units (OTUs) clustering. Then, clustering was performed based on the clean tag, the chimera tags detected in the cluster comparison process were removed, and the naïve Bayesian assignment algorithm of the RDP Classifier was applied to the representative sequences. Finally, the species were annotated using the database (confidence threshold set to 0.8–1).

### 2.4 Statistical analysis

SPSS software was used to perform multivariate analysis of variance (ANOVA) to test the significant differences of OTU number and bacterial α-diversity indices among the treatments. All data were expressed as mean ± standard error. The Shapiro–Wilk test was used to assess whether the data were normally distributed (*P* > 0.05). The Kruskal–Wallis rank sum test model was used to identify whether there were differences in the median species abundances among treatment groups. Origin 2019b and Excel 2010 software were used for illustrations and tables, respectively.

## 3. Results

### 3.1 Comparative analysis of growth under the three regeneration methods

A comparison of the growth under the three regeneration methods (Table 2) showed that there were no significant differences in tree height and DBH between ST and SP in 2017 or 2020, but both tree height and DBH were significantly higher in ST and SP than in CK. Compared with 2017, tree height in CK was 38.11% higher in 2020 and DBH was 26.62% larger. In ST, tree height increased by 41.02% and DBH increased by 39.38%. In SP, tree height increased by 41.64% and DBH increased by 38.28%. Thus, SP had the greatest increase in tree height, while ST had the largest increase in DBH.

**Table 2:** Growth indices of poplar 107 under the three regeneration methods in different years.

Years	Regeneration methods	Height of tree(m)	DBH(cm)
2017	CK	7.82±0.41b	10.82±0.39b
	ST	8.24±0.29a	11.30±0.56a
	SP	8.14±0.27a	11.26±0.46a
2020	CK	10.80±0.39b	13.70±0.58b
	ST	11.62±0.74a	15.75±1.04a
	SP	11.53±0.58a	15.57±1.48a

Note: The same lowercase letters in each column indicate that the difference did not reach a significant level in the same year (*P* > 0.05).

### 3.2 Operational taxonomic unit (OTU) number and bacterial community diversity

**Fig 1:**
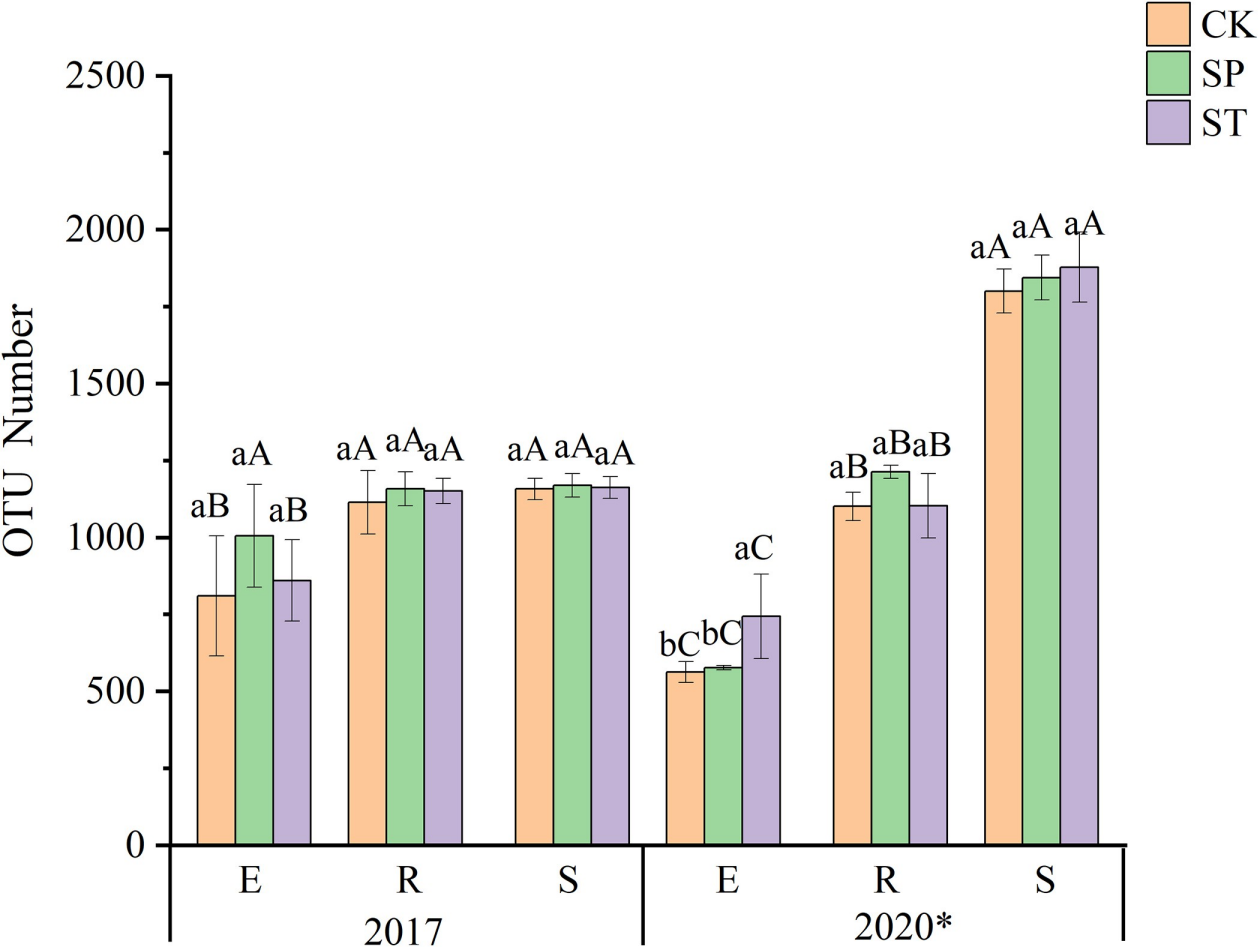
OTU number for the three regeneration methods. Various lowercase letters indicate significant differences between different regeneration methods in the same year and the same sample type (*P* < 0.05), different uppercase letters indicate significant differences between different sample types with the same regeneration method in the same year (*P* < 0.05), and * indicates significant differences between years (*P* < 0.05).

Analysis of the OTU number (Fig 1) showed that the number of OTUs in root and rhizosphere soil samples of ST and CK was significantly higher than in stem samples in 2017, but there was no significant difference among these three sample types for SP. In 2020, the number of OTUs in rhizosphere soil was significantly higher than in roots and stems.

Comparison among the three regeneration methods showed that the number of OTUs in SP was higher than in ST or CK in 2017, but the differences among the three regeneration methods were not significant. In 2020, the number of stem endophytic bacterial OTUs was significantly higher in ST than in SP or CK. There were also significant differences in the number of OTUs between the two years. The results showed that ST increased the number of stem endophytic bacterial OTUs, while the number of OTUs had little difference between SP and CK. Among the three sample types, the number of the underground bacterial OTUs was higher than the number of aboveground bacterial OTUs. With the increase in stand age, the number of OTUs increased.

**Fig 2:**
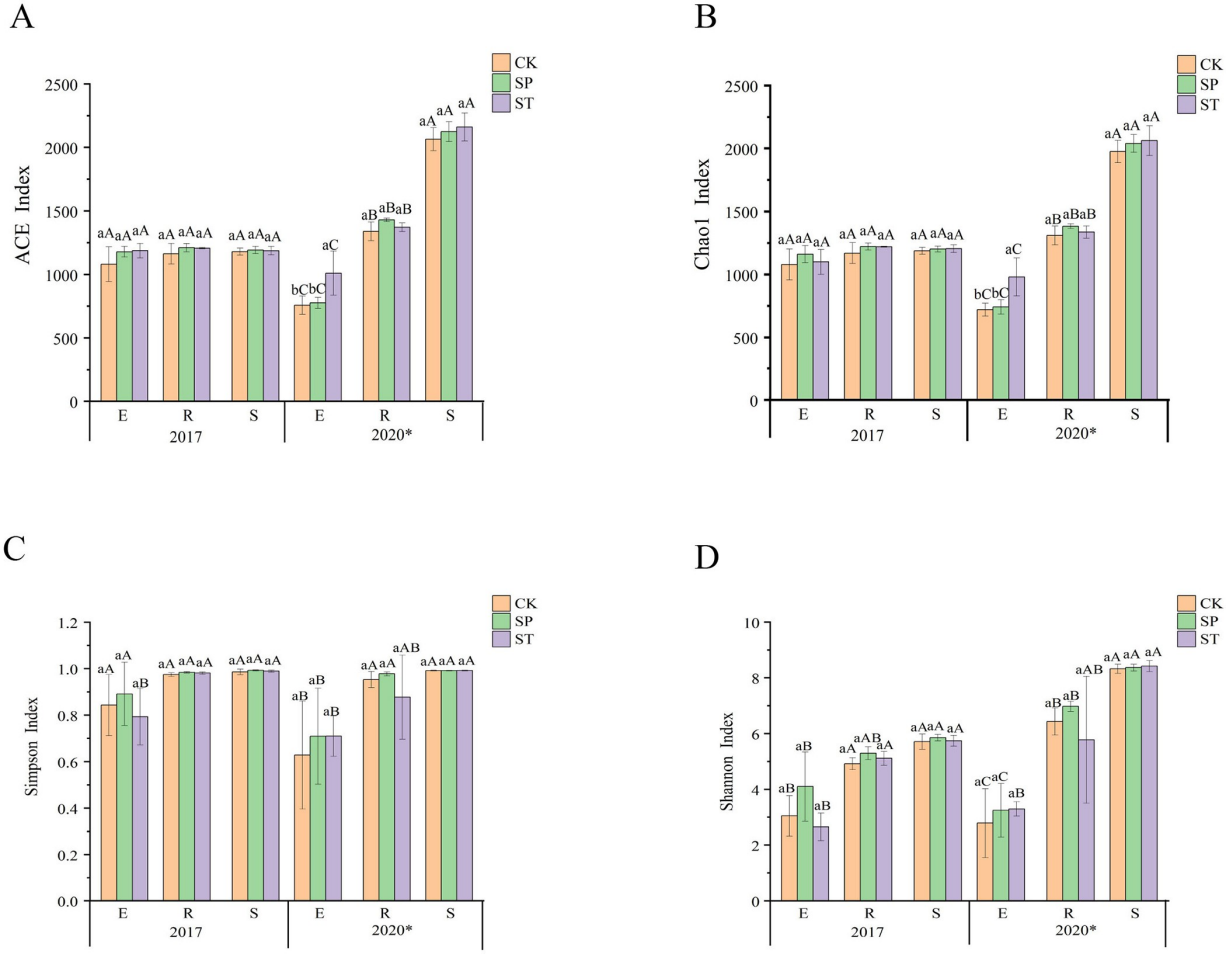
Bacterial α-diversity indices of the three regeneration methods. (A) ACE index, (B) Chao1 index, (C) simpson index and (D) Shannon index. Various lowercase letters indicate significant differences between different regeneration methods in the same year and the same sample type (*P* < 0.05), different uppercase letters indicate significant differences between different sample types with the same regeneration method in the same year (*P <* 0.05), and * indicates significant differences between years (*P* < 0.05).

The richness and diversity of ecosystem species are typically evaluated using α-diversity indices. The ACE and Chao1 indices reflect the species diversity of samples, with larger values indicating higher diversity. Additionally, higher Simpson and Shannon index values reflect higher species richness and evenness. The analysis of the ACE and Chao1 indices of poplar 107 (Fig 2) showed that there were no significant differences among the three sample types in 2017. In 2020, the rhizosphere soil samples had significantly higher values than the root samples and the stem samples. In 2017, there were no significant differences between the three regeneration methods. However, in 2020, the ACE index and Chao1 index of stem endophytic bacteria were significantly higher in ST than in SP or CK. These two indices of root and rhizosphere soil samples were slightly higher in SP and ST than in CK, but the differences were not significant.

The Simpson index and Shannon index showed no significant differences among CK, SP, and ST. There were no significant differences in the Simpson index between root and rhizosphere soil, but these values were significantly higher than the value for stem. The Shannon index value of rhizospheric bacteria was significantly higher than the index values for root and stem endophytic bacteria. The four indices showed significant differences between the two years. The results showed that the diversity of stem endophytic bacteria was significantly higher in ST than in SP or CK and that the rhizosphere bacterial diversity was slightly higher in SP and ST than in CK. The differences in bacterial richness and evenness among the three regeneration methods were small. However, different years and sample types had greater impacts on bacterial diversity, richness, and evenness.

**Fig 3:**
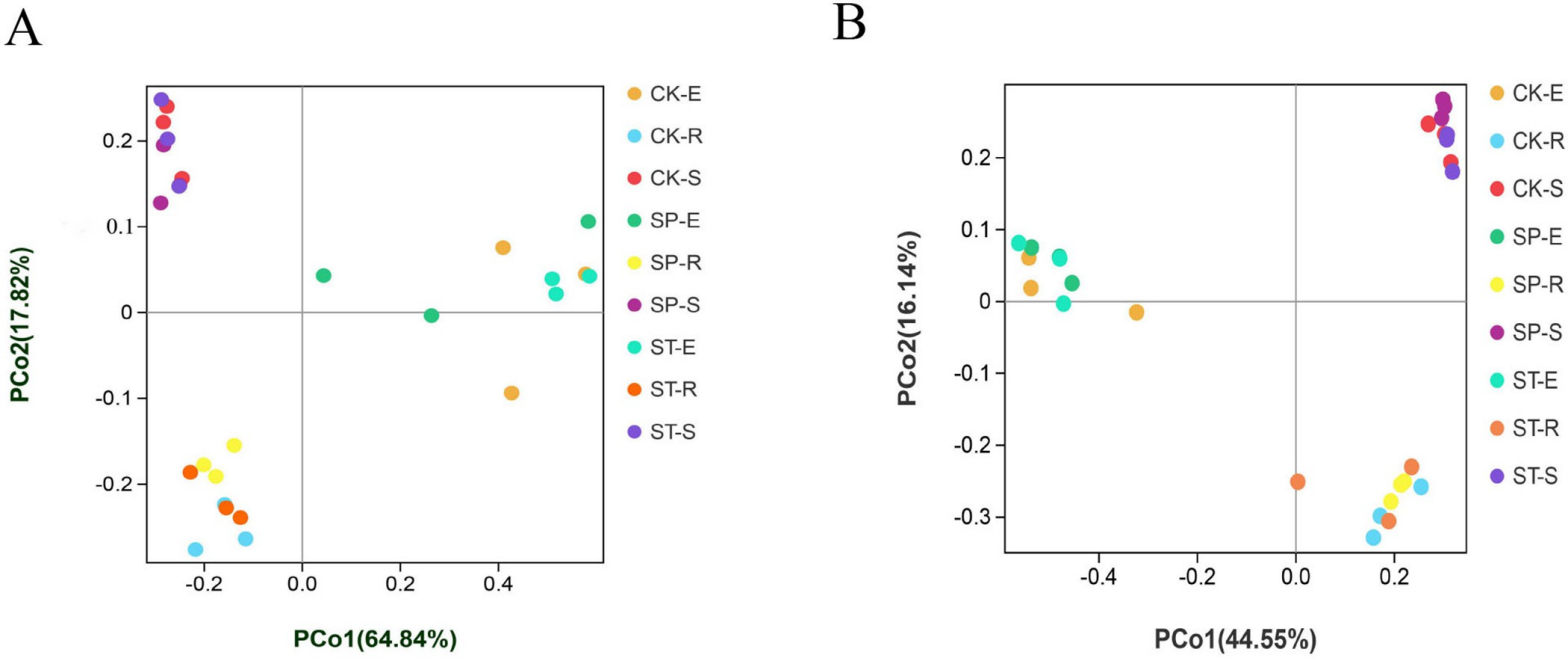
PCoA analysis of the bacterial community based on Bray–Curtis distance. (A) PCoA analysis for 2017 and (B) PCoA analysis for 2020.

Cluster analysis of bacterial OTUs based on Bray–Curtis distance (i.e., PCoA) was used to evaluate the similarity of bacterial community composition. The cumulative variance contribution of the first two principal components extracted in 2017 was 82.66%, which accounted for 64.84% and 17.82% of the variance of the variables, respectively (Fig 3A). In 2020, the cumulative variance contribution of the two principal components was 60.69%, which explained 44.55% and 16.14% of the variance of the variables, respectively (Fig 3B). In the two years, the same sample types clustered together, and there was an obvious separation among the three sample types. The long distance among stem samples indicated that the stem endophytic bacterial community had low similarity. There was no obvious clustering among the three regeneration methods. The results showed that the bacterial community structures in the three regeneration methods were similar but varied greatly among sample types.

**Fig 4:**
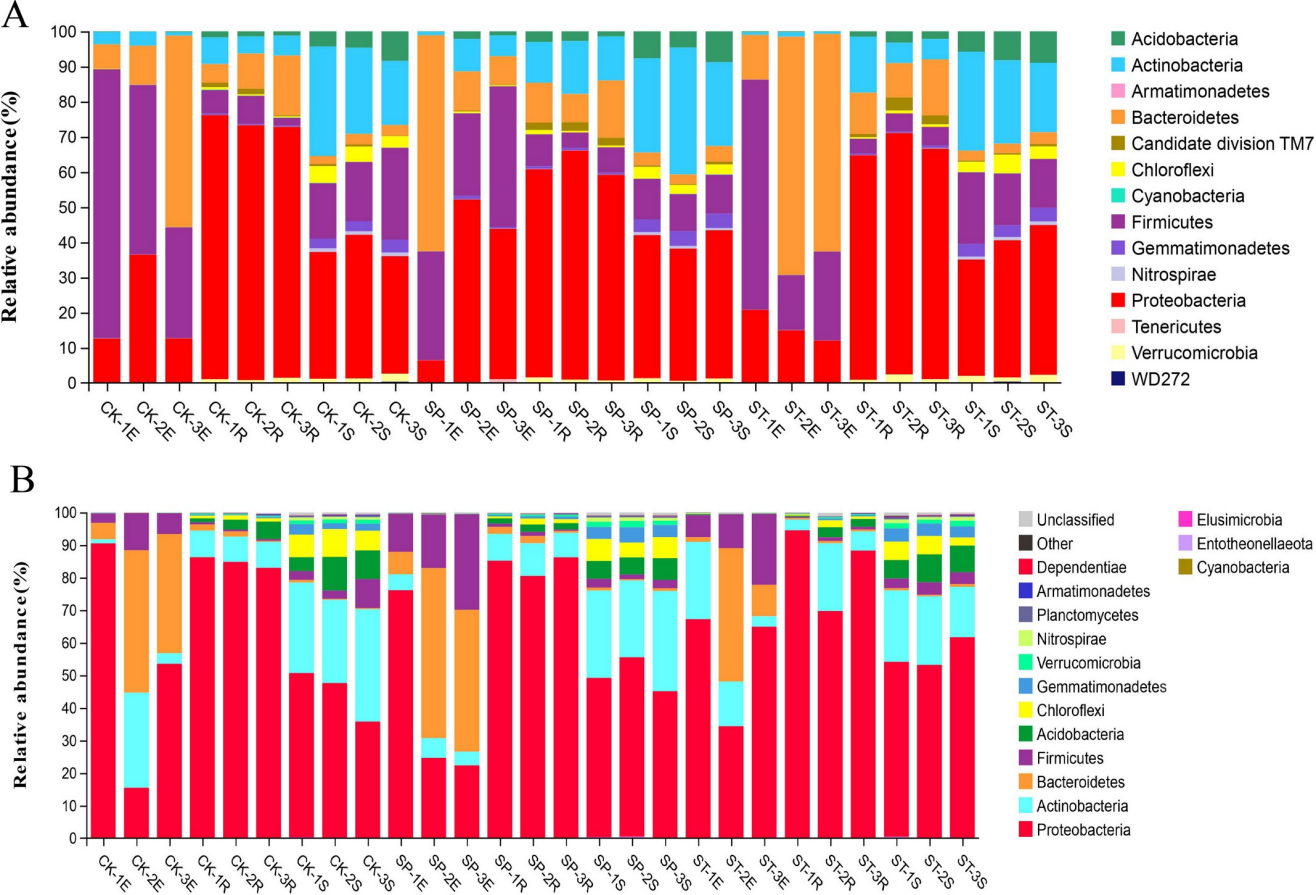
Relative abundances of bacterial communities at the phylum level under the three regeneration methods. A and B represent the relative abundances of bacterial communities in 2017 and 2020, respectively.

The relative abundances of species at the phylum level are shown in a stacking diagram (Fig 4). In 2017, 14 bacterial phyla, in total, were annotated. In 2020, 27 bacterial phyla were annotated (only the top 15 species based on relative abundance are shown in the figure). A comparison of the two years showed that 11 phyla were in common, namely, Proteobacteria, Firmicutes, Acidobacteria, Actinobacteria, Bacteroidetes, Chloroflexi, Cyanobacteria, Gemmatimonadetes, Nitrospirae, Verrucomicrobia, and Armatimonadetes. The unique groups in 2017 were Candidate Division TM7, WD272, and Tenericutes. In 2020, Planctomycetes, Dependentiae, Elusimicrobia, and Entotheonellaeota were added to the top 15 species by relative abundance. In 2017, the abundances of Proteobacteria in CK, SP, and ST were 43.47%, 44.96%, and 40.07%, respectively. In 2020, these abundances were 60.75%, 58.16%, and 65.20%, respectively. In 2017, the abundances of Acidobacteria in CK, SP, and ST were 3.78%, 3.54%, and 3.41%; in 2020, they were 3.67%, 2.60%, and 3.20%, respectively.

The dominant phyla of stem endophytic bacteria were Proteobacteria, Firmicutes, and Bacteroidetes, which accounted for 92.71% of the total. Proteobacteria and Actinobacteria were dominant in roots, accounting for 84.59% of the total. The dominant bacteria in rhizosphere soil were Proteobacteria, Acidobacteria, Actinobacteria, and Firmicutes, accounting for 86.28% of the total. Most bacteria exhibited significant differences between the sample types. For example, the relative abundance of Proteobacteria was significantly higher in the root samples than in the stem or rhizosphere soil samples. The abundances of Actinobacteria and Acidobacteria were significantly higher in rhizosphere soil than in roots or stems. The abundances of Firmicutes and Bacteroidetes were significantly higher in stems than in roots or rhizosphere soil. Comparison of the three regeneration methods showed that the dominant flora were basically the same in each method. They mainly included Proteobacteria, Bacteroidetes, Firmicutes, Actinobacteria, and Acidobacteria. In 2017, these bacterial taxa accounted for 95.42%, 94.87%, and 94.59% of the flora in CK, ST, and SP, respectively. In 2020, they accounted for 94.72%, 94.69%, and 93.95%, respectively. The relative abundance of the dominant bacterial taxa decreased with increased stand age.

**Fig 5:**
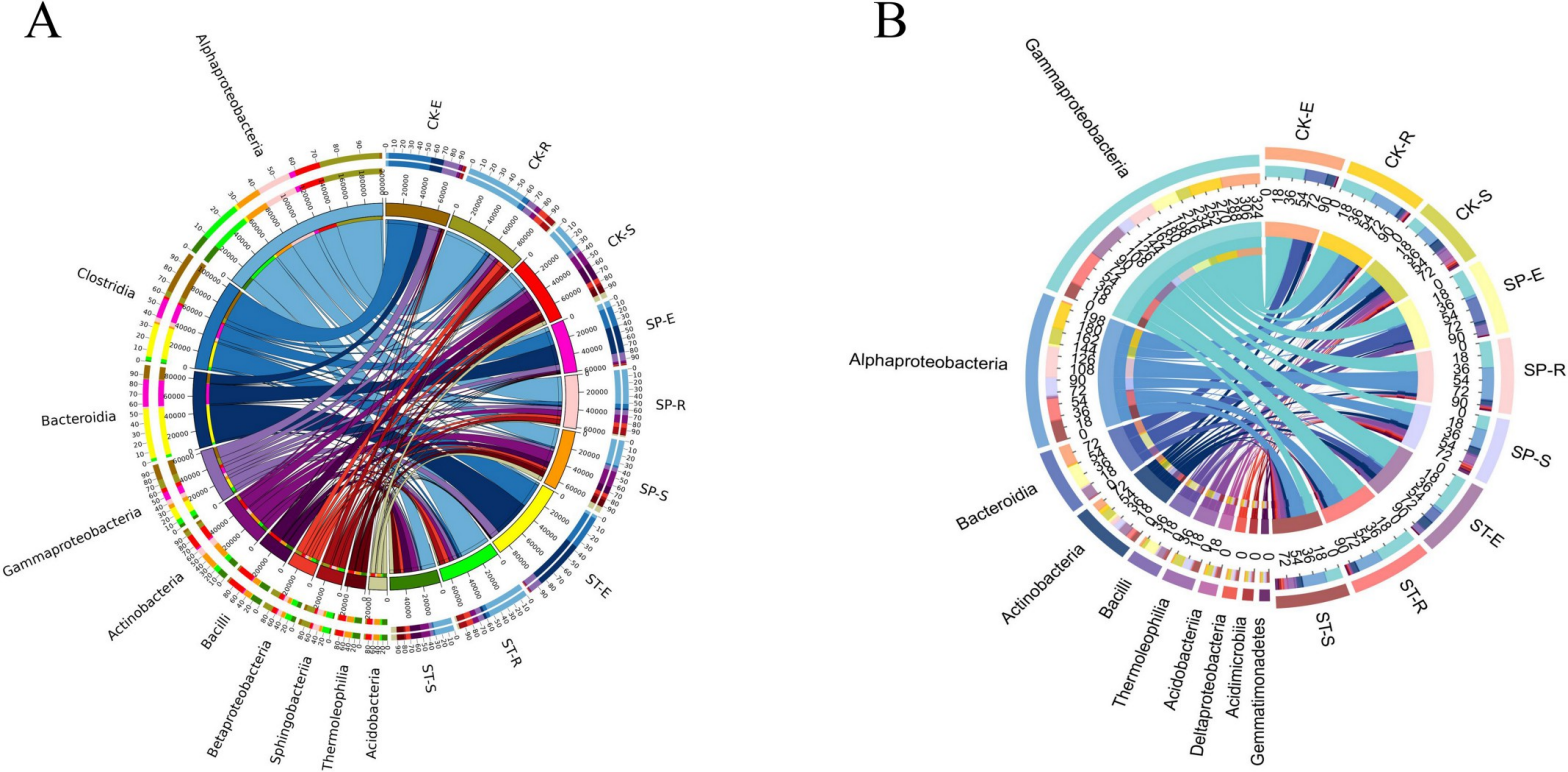
Circos diagram of the composition and distribution ratio of the dominant bacteria under the three regeneration methods. A and B represent the composition and distribution ratio of dominant bacteria in 2017 and 2020, respectively.

**Fig 6:**
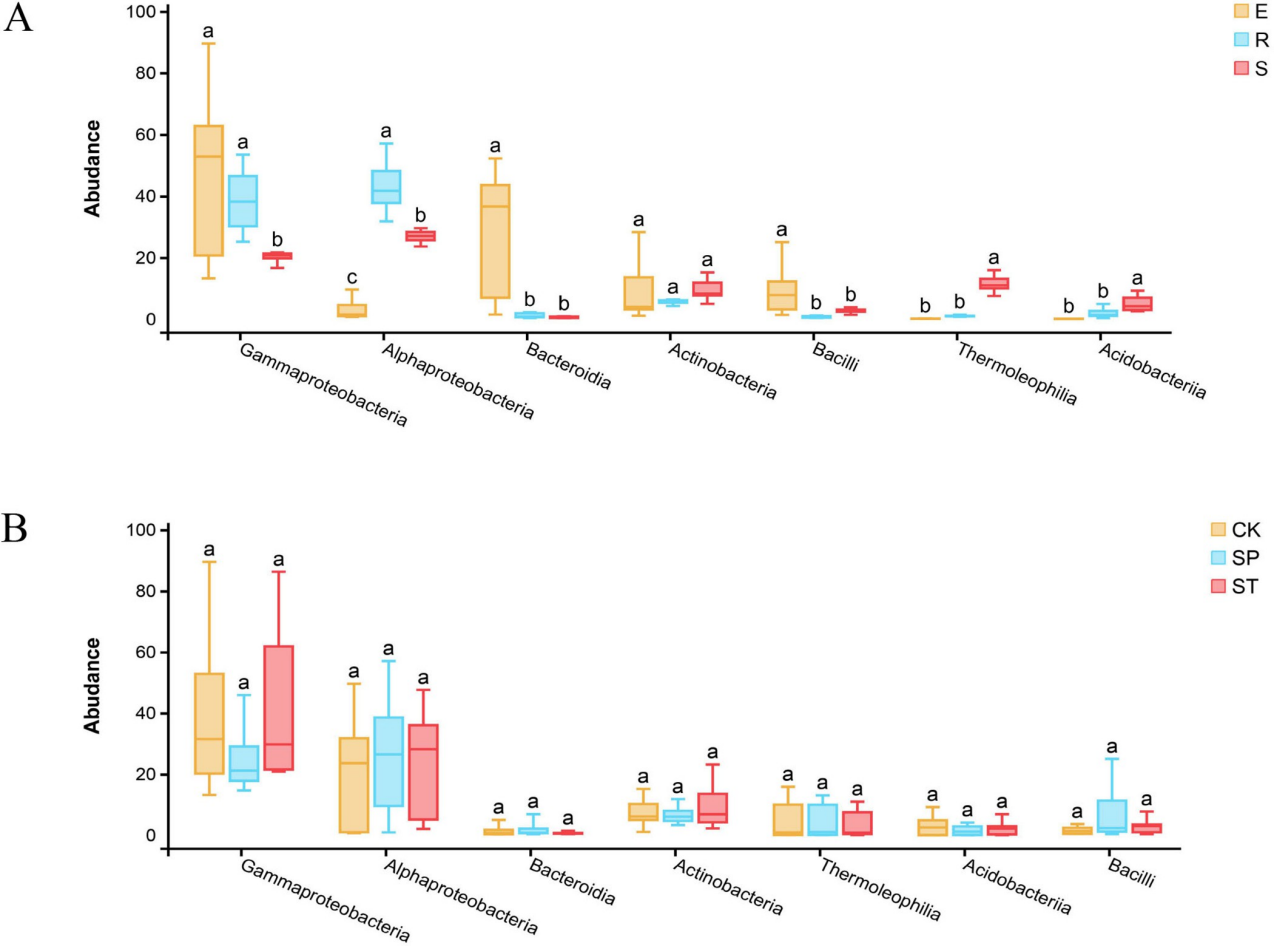
Differences in bacterial community composition at the class level. (A) Tukey HSD test under the three sample types and (B) Tukey HSD test under the three regeneration methods.

The bacterial composition and distribution ratio (top 10 in abundance) at the class level is shown in a Circos diagram (Fig 5). A comparison of the two illustrations shows that 7 among the top 10 classes were the same in the two years: Alphaproteobacteria, Bacteroidia, Gammaproteobacteria, Actinobacteria, Bacilli, Thermoleophilia, and Acidobacteriia. The Tukey HSD test was performed on the seven taxa shared in the two years under the three sample types and the three regeneration methods. The abundances of the seven bacterial taxa showed significant differences in the sample types (Tukey HSD: *P* < 0.05) (Fig 6A). Alphaproteobacteria (E: 2.91%, R: 38.97%, S: 26.95%) were significantly enriched in root samples. The abundance of Gammaproteobacteria (E: 46.59%, R: 43.65%, S: 19.32%) was significantly higher in plant tissues (stems and roots) than in rhizosphere soil. Bacteroidia (E: 26.65%, R: 1.15%, S: 0.54%) and Bacilli (E: 9.43%, R: 0.64%, S: 3.29%) were enriched in stem samples. Thermoleophilia (E: 0.12%, R: 1.13%, S: 11.37%) and Acidobacteriia (E: 0.06%, R: 1.89%, S: 5.14%) were significantly higher in rhizosphere soil samples than in root and stem samples. Among the three regeneration methods (Fig 6B), the results showed no significant differences in abundance (*P* > 0.05) of the seven taxa. These results indicated that the compositions and proportions of bacteria varied greatly among the two years and three sample types, but that the three regeneration methods had little effect on the bacterial community structure.

### 3.3 Differences in plant growth promoting rhizobacteria (PGPR) abundances among the three regeneration methods

**Fig 7:**
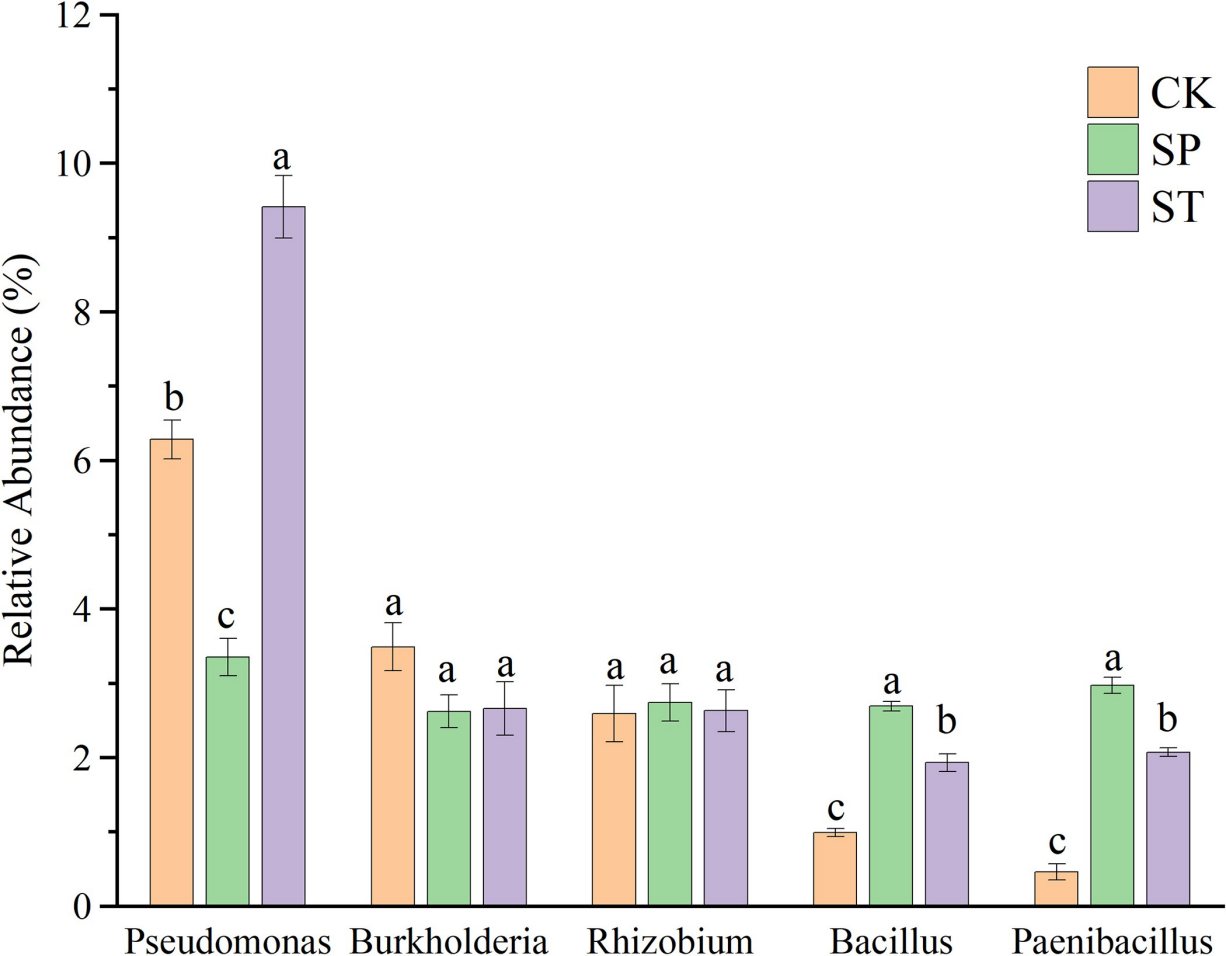
Differences in PGPRs abundance under the three regeneration methods.

PGPRs are beneficial rhizosphere bacteria that have a positive effect on plant growth. At present, a variety of PGPR strains are used commercially. These include *Azospirillum*, *Bacillus*, *Burkholderia*, *Paenibacillus*, *Pseudomonas*, *Rhizobium*, *Serratia*, and more [18]. The relative abundances of the PGPRs in this study were compared among the three regeneration methods (Fig 7). The abundances of most taxa were higher in SP and ST than in CK. The abundances of *Bacillus* and *Paenibacillus* were significantly higher in SP than in ST or CK. The abundance of *Pseudomonas* was significantly higher in ST than in SP or CK. *Rhizobium* and *Burkholderia* showed differences among the three regeneration methods, but these differences were not significant. Among these five bacteria, only *Burkholderia* had its highest abundance in CK. This indicates that more PGPRs were present in the rhizospheres of SP and ST trees.

### 3.4 Analysis of the differential bacteria under the three regeneration methods

**Fig 8:**
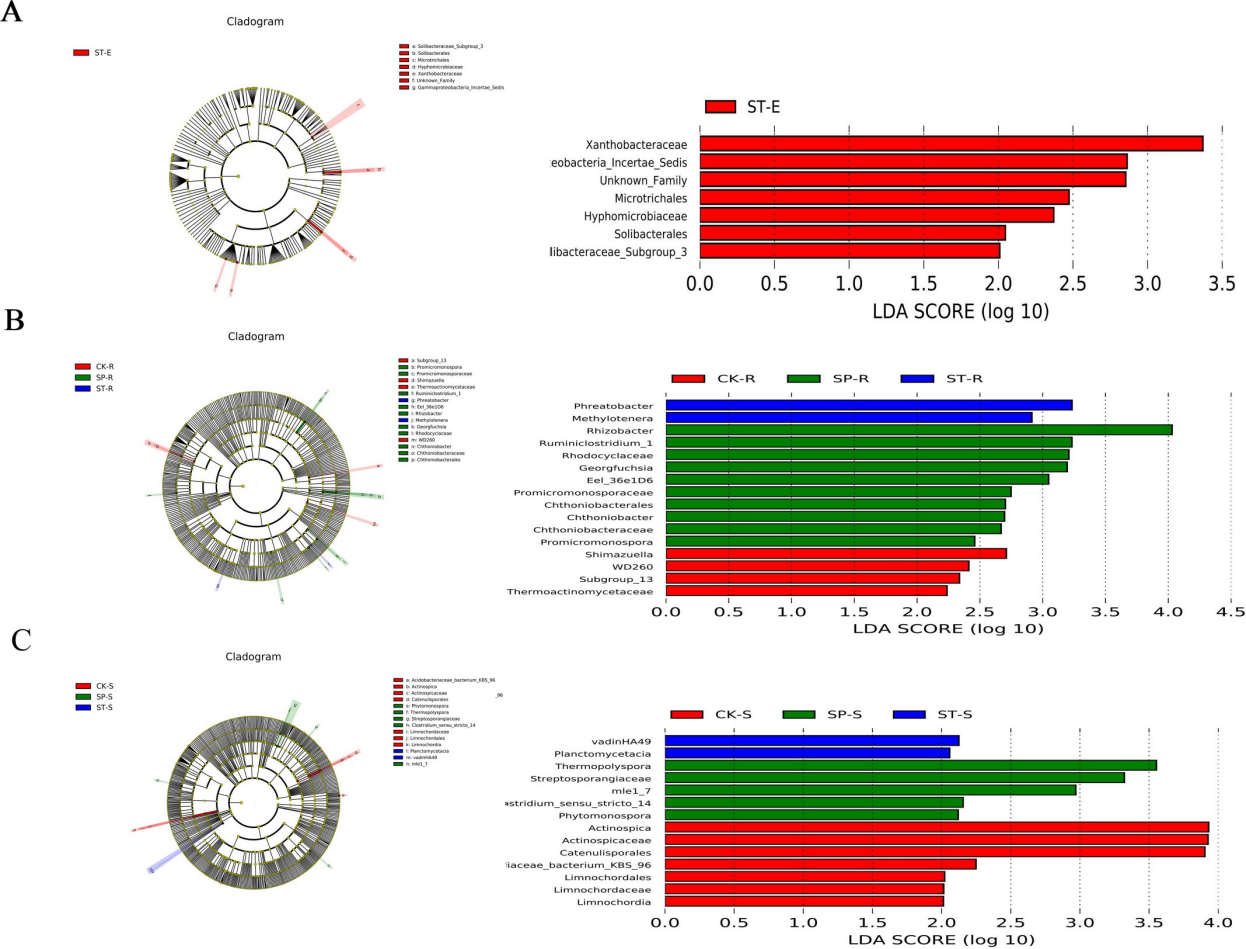
**Cladogram (left) and LDA histogram (right) of the three regeneration methods.** (A) Stem endophytic differential bacteria, (B) root endophytic differential bacteria and (C) rhizophere soil differential bacteria.

LEfSe analysis revealed the differences in bacteria distribution from phylum to genus among the three regeneration methods. A cladogram is a classification tree that maps differences to a known hierarchical structure. The length of the histogram represents the impact of different species (i.e., LDA score), and the influence is displayed when the LDA value exceeds 2. Among the stem endophytic bacteria samples, there were seven different species, all in ST, belonging to three orders and four families (Fig 8A). The most influential taxon was Xanthobacteraceae, which belongs to the α-Proteobacteria. The rest included Microtrichales, Solibacterales, Solibacteraceae_Subgroup_3, Gammaproteobacteria_Incertae_Sedis, Hyphomicrobiaceae, and Unknown_Family.

In the root samples, 16 different bacteria were identified (Fig 8B). The specific taxa in CK included Subgroup_13 and WD260 at the order level, Thermoactinomycetaceae at the family level, and *Shimazuella* at the genus level. There were 10 indicator taxa in SP: Chthoniobacterales at the order level; four taxa at the family level, namely, Eel_36e1D6, Chthoniobacteraceae, Rhodocyclaceae, and Promicromonosporaceae; and five at the genus level, *Promicromonospora*, *Rhizobacter*, *Ruminiclostridium_1*, *Chthoniobacter*, and *Georgfuchsia*. The different taxa in ST were *Phreatobacter* and *Methylotenera*, both at the genus level.

In the rhizosphere soil samples, there were 14 different taxa (Fig 8C). The different taxa in CK were *Actinospica* (from family to genus) and Catenulisporales, Acidobacteriaceae_bacterium_KBS_96, and Limnochordaceae (from class to family), which belong to three phyla. Among these, *Actinospica* (from family to genus) and Catenulisporales, all belonging to the Actinobacteria, had the greatest influence. There were five different taxa in SP, namely, Streptosporangiaceae, *Thermopolyspora*, *Phytomonospora*, *mle1_7*, and *Clostridium_sensu_stricto_14*, which belong to three phyla. ST had two different taxa, vadinHA49 and Planctomycetacia, both belonging to the Planctomycetes.

## 4. Discussion

### 4.1 Effects of the three regeneration methods on the growth of poplar 107

Because poplar is the main fast-growing plantation tree species in China, it is important to study effective regeneration methods for its rapid and healthy growth. The three different regeneration methods examined in this study had different effects on poplar 107 growth. In a comparison of the height and DBH of trees in 2017 and 2020, SP and ST produced significantly higher values than CK, possibly because the SP and ST methods made use of the nutrients in the stumps of the perennial mother trees to grow rapidly and also because they accelerate the absorption of water and nutrients by the trees. Stumps have the characteristic of slow release of nutrients, which can provide steady nutrients for growth of the forest [19]. The same results have been obtained by previous studies. Li et al. [3] found more rapid growth in stump grafting than in planted seedling afforestation. Zhang et al. [20] observed the survival rate and growth of stump sprouting regeneration and planted seedling afforestation of poplar I-69. The results showed significantly higher tree height and DBH under stump sprouting regeneration than under planted seedling afforestation. In addition, Shimaila et al. [21] showed that PGPRs, such as *Bacillus* and *Pseudomonas*, can promote plant growth by expressing 1-aminocyclopropane- 1-carboxylate (ACC) deaminase to decompose the ethylene precursor ACC, thereby reducing potentially harmful levels of ethylene in plant hosts. However, in this study, most of the PGPRs were enriched in SP and ST, which may explain the enhanced growth under SP and ST.

### 4.2 Bacterial community structure and diversity in the three sample types

Microorganisms play an important role through their symbiosis with plants. The complex nutritional environment in plants determines the different bacterial communities that become established in different plant organs [22]. Soil bacteria are the most abundant and widely distributed microbial group in soil, and they play an important role in biogeochemical cycles [17]. Plants influence rhizosphere soil microbial communities through leaf litter and root secretions. In turn, the biological traits of microorganisms affect the host plants and their coexisting plants [23]. In this study, we found that the bacterial diversity was significantly higher in rhizosphere soil samples than in root endophytic bacteria or stem endophytic bacteria samples. This is consistent with the results of Durand et al. [24] and Knief et al. [25], who also found that the aboveground bacterial community had lower richness and diversity than the underground community in their study of bacterial community composition of poplar and rice. The PCoA analysis (Fig 3) showed that the root and rhizosphere soil samples had relatively small differences in bacterial communities but that the stem endophytic bacterial communities varied greatly, which is consistent with the results of Beckers et al. [26]. This result may be due to the different properties and abilities of the endophytic bacteria that colonize plants (e.g., the motility of bacteria, their ability to produce cell wall degrading enzymes) [27, 28] and their interaction with the innate immune system of host plants [29]. However, the formation of the rhizosphere bacterial community mediated by carbon deposition (root exudates, etc.) and the transfer of photosynthates is a consistent and stable process in poplar [26].

Yang et al. [30] found that Bacteroidetes and Firmicutes were more abundant in endophytic communities in leaves than in roots. Our results aresimilar. The abundances of Bacteroidetes and Firmicutes were higher in stems than in roots, which might be related to community niche preference. In addition, we also found that Acidobacteria were significantly enriched in rhizosphere soil. Alphaproteobacteria was the dominant phylum in roots, and the abundance of Gammaproteobacteria was significantly higher in plant tissues (stems and roots) than in rhizosphere soil. This result is similar to the results of other studies on poplar [31, 32], Arabidopsis [33, 34], and rice [35]. The results showed that the relative abundances of Acidobacteria and Actinobacteria decreased from rhizosphere soil to roots. The relative abundance of Proteobacteria (mainly Alphaproteobacteria) was high in roots. Additionally, stem and leaf endophytic bacteria were dominated by Proteobacteria, most of which belonged to Gammaproteobacteria.

### 4.3 Effects of stand age on bacterial community structure and diversity

We found that the four diversity indices of bacteria increased significantly with increased stand age. This result is consistent with the results of Liu et al. [36], which showed that with increased stand age, human disturbance decreased, and the understory vegetation growth and humus accumulation contributed to the growth of the microbial population. The dominant taxa of the stand were the same in the two years and mainly included Proteobacteria, Bacteroidetes, Firmicutes, Actinobacteria, and Acidobacteria. Compared with 2017, the relative abundance of dominant taxa was lower in 2020. Liang et al. [37] found the same result. This may occur because a decrease in the abundance of dominant species is conducive to the growth of non-dominant species, which increases microbial diversity.

Proteobacteria, as the dominant bacteria with the highest abundance in soil, can respond rapidly to unstable carbon sources and grow rapidly in a carbon-rich environment [38]. With increased stand age, the abundance of Proteobacteria increased significantly, as also shown by Carson and Zeglin [39]. It may be that carbon and nitrogen sources in the soil increase with stand age, causing Proteobacteria to also increase. Acidobacteria is an oligotrophic bacterium, and its relative abundance tends to be higher when the soil carbon concentration is low [40, 41]. The abundance of Acidobacteria decreased with greater forest age, and the abundance was lower in SP and ST than in CK. This result may be due to the increased age of the stand leading to an increase in the amount of litter returned to the soil and also an increase in soil nutrients, resulting in a decrease in the abundance of Acidobacteria. Studies have shown that the non-structural substances (leaves, fine roots) in litter have a higher decomposition rate and faster release rate of nutrients in the early stage [42]. However, it takes years or even decades for C and N to be released from structurally complex residues such as tree stumps and thick roots [43]. The reason the abundance of Acidobacteria was lower in ST and SP than in CK may be that the C and N in the structurally complex residues were released gradually over time, which increased the nutrient input to the soil and caused nutrient enrichment in the rhizosphere soil of ST and SP, leading to low abundances of Acidobacteria.

### 4.4 Effects of regeneration methods on bacterial community structure and diversity

Fast-growing and high-yielding poplar forests are generally harvested every 5–7 years, and effective regeneration methods are one of the important ways to promote rapid and healthy establishment of poplar forests. Few studies have assessed whether different regeneration methods affect bacterial diversity. The OTU number and alpha diversity index (Figs 1 and 2) evaluations in this study showed that there were no significant differences among the three regeneration methods. This may be because many bacteria do not depend on their symbiotic hosts and have a high tolerance and flexibility to soil environmental variations, resulting in their reduced sensitivity [44]. However, ST significantly increased the diversity of stem endophytic bacteria, which may be due to the communication and transmission of bacteria between the scion and the rootstock caused by grafting, thus increasing the diversity of ST stem endophytic bacteria. Johnston-Monje and Raizada [45] found that green fluorescent protein-labeled endophytes could be transported from seeds to roots and stems and that endophytes could also migrate from stems to roots and even the rhizosphere to improve the condition of microorganisms. The existence of the mother tree is the basis for the emergence of the sprouting phenomenon [46]. The sprouting seedlings continue to grow and develop on the stumps, and there is a balance of nutrients in the underground and aboveground. The root system of the mother tree can provide a source of nutrients for the initial growth of the sprouting stem in the early stage [47]. At the same time, the aboveground part receives light to synthesize carbohydrates, but the continued growth and development of the stump need to absorb nutrients, which may affect the growth of the sprouting stem [48]. Therefore, we used the method of sealing soil to make the stumps rot, so that the seedlings could rebuild the root system. Root rot promotion is an important measure to promote the decomposition of stumps and improve the productivity of stand. In this study, we found that *Chthoniobacter* was significantly enriched in SP (Fig 8B), *Chthoniobacter* has the ability to degrade complex organic compounds [49], which may have played an important role in the process of root decay and decomposition. *Bacillus* significantly enriched in SP can enhance nutrients uptake by plants [50]. This suggests that some microorganisms promote the process of root rot and decomposition, organic matter production and utilization by new plants.

As a major component of forest residues, stump provides habitat for microorganisms [51]. Garbelotto et al. [52] showed that disease severity tends to be higher in frequently felled and thinned forests and in older forests. Due to the large and far-reaching root system of old trees, it is more conducive to extensive secondary transmission, and the secondary roots of stumps are also more likely to colonize some pathogenic bacteria. In this study, the relative abundance of Actinobacteria was significantly higher in CK than in SP and ST (Fig 8C). Studies have found that Actinobacteria are able to resist pathogen infection [53]. This result may have occurred because some tree pathogens can survive in large dead roots and tree stumps [52], and the Actinobacteria in ST and SP aided the plants in removing pathogens from their tissues, resulting in a decrease in their content.

Therefore, while using stump grafting and stump sprouting for regeneration of forest land, it is also necessary to prevent and control the harm of toxic substances and pathogenic microorganisms that may be produced during the decomposition of tree stumps to the growth of the next generation seedlings. Most root-disease microorganisms can survive for decades in large tree stumps [54], and when logging is used for long-term regeneration, we need to take effective forest management measures to understand the biology of pathogenic microorganisms, limit their establishment and spread, and log tree stumps when necessary to reduce pathogen damage to minimize the level of infection [55, 56].

This study also found that specific bacterial species were associated with the three different regeneration methods (Fig 8). Some studies have revealed that plant genotype is an important determinant of bacterial community composition [57, 58]. A second factor is the plants’ own needs to grow. Many different species of plants, and even the same species growing in the same environment, also have a unique group of bacteria [59–61]. Overall, our study indicated that the three regeneration methods had little effect on microbial community structure, but had an impact on bacterial composition; Because our research focused only on 4- and 7-year-old poplar 107, additional generations of regeneration and longer-term microbial community monitoring need to be implemented.

## 5. Conclusion

Investigation of tree growth under the three regeneration methods in two years proved that stump grafting and stump sprouting regeneration are more conducive to growth of poplar 107 than planted seedling regeneration. Based on bacterial 16S rDNA sequencing, we found that the number of OTUs and the alpha diversity indices did not differ significantly among the three regeneration methods. Only the stem endophytic bacterial diversity of ST was higher than the diversity of CK and SP. The diversity and community composition of the bacteria varied greatly among years and sample types. The sample type and stand age were the main factors driving changes in the poplar 107 bacterial community, and the three regeneration methods had little effect on the community. Thus, regeneration and transformation of poplar plantations can be better carried out by stump grafting and stump sprouting.

## References

[1] Navarro A, Facciotto G, Campi P, Mastrorilli M. Physiological adaptations of five poplar genotypes grown under SRC in the semi-arid Mediterranean environment. Trees-Struct Funct. 2014; 28(4): 983–994.

[2] Wu LJ. Benefit analysis of regeneration and transformation of poplar low-efficiency forest by stump grafting. Forest Resour Manag. 2013; 5: 47–51.

[3] Li GH, Wu LJ, Zhang YY, Dang HZ, Wu XL, Zhou ZF, et al. Comparison of growth and carbon storage of poplar stump grafting and seedling forest. Forest Res. 2013; 26(06): 800–804.

[4] Bond WJ, Midgley JJ. The evolutionary ecology of sprouting in woody plants. Int J Plant Sci. 2003; 164(S3): S103–S114.

[5] Xu J, Huang DZ, Wen J, Wang LH. Anatomical characteristics of the wood of stump grafted *Populus tomentosa*. J Northeast Forestry Univ. 2014; 42(08), 82–85 + 89.

[6] Geng B, Wang HT, Wang YP, Xue BJ, Li WQ. Comparison of growth between *Robinia pseudoacacia* coptic forest and seedling forest. Sci Soil Water Conserv. 2013; 11(02): 59–64.

[7] Kammesheidt L. The Role of tree Sprouts in the Restoration of Stand Structure and Species Diversity in Tropical Moist Forest after Slash-and-Burn Agriculture in Eastern Paraguay. Plant Ecol. 1998; 139, 155–165.

[8] Lv DG, Gao H, Qin SJ, Liu LZ, Ma HY. Effects of grafting on the structure and diversity of rhizosphere microbial community of *Cerasus sachalinensis* Kom. Chinese Agr Sci B. 2011; 27(10): 82–87.

[9] Feng YJ, Song W. Plant endophytic bacteria. J Nature. 2001; 5, 249–252.

[10] Ravin P, Ari J, Megan MK, Cary LR, Lorena GM, Karen AG. Rootstocks Shape the Rhizobiome: Rhizosphere and Endosphere Bacterial Communities in the Grafted Tomato System. Appl Environ Microb. 2019; 85(2): 1–16. 10.1128/AEM.01765-18 PMC6328775 30413478

[11] Yang J, Dong CB, Zhang ZY, Liang JD, Han YF, Liang ZQ. Diversity analysis of fungal community structure in rhizosphere soil of *Eucommia ulmoides* from different habitats. Acta Mycol Sinica. 2019; 38(3): 327–340.

[12] Li H, Su JQ, Yang XR, Zhu YG. Distinct rhizosphere effect on active and total bacterial communities in paddy soils. Sci. Total Environ. 2019; 649: 422–430. doi: 10.1016/j.scitotenv.2018.08.373 30176455

[13] Hacquard S, Garrido-Oter R, Gonzalez A, Spaepen S, Ackermann G, Lebeis S, et al. Microbiota and host nutrition across plant and animal kingdoms. Cell Host & Microbe, 2015; 17(5): 603–616. 10.1016/j.chom.2015.04.009 25974302

[14] Mishra PK, Bisht SC, Mishra S. Coinoculation of rhizobium Leguminosarum-Pr1 with a cold tolerant pseudomonas sp. improves iron acquisition, nutrient uptake and growth of field pea (*Pisum Sativuml*.). J Plant Nutr. 2012; 35(2): 243–256.

[15] Bulgarelli D, Schlaeppi K, Spaepen S. Structure and functions of the bacterial microbiota of plants. Annu. Rev. Plant Biol. 2013; 64, 807–838. doi: 10.1146/annurev-arplant-050312-120106 23373698

[16] Yang J, Kloepper JW, Ryu CM. Rhizosphere bacteria help plants tolerate abiotic stress. Trends Plant Sci. 2009; 14(1): 1–4. doi: 10.1016/j.tplants.2008.10.004 19056309

[17] Liu WS, Li HQ, He Y, Huang YY, Qiu KY, Xie YZ. Research progress of rhizosphere microorganisms on the interaction between plants and soil. Soil Fertil Sci China. 2021; 5, 1–14.

[18] Glick BR. Plant growth promoting bacteria: mechanisms and applications. Scientifica. 2012; 1–15. doi: 10.6064/2012/963401 24278762 PMC3820493

[19] Persson T. Environmental consequences of tree- stump harvesting. Forest Ecol Manag. 2013; 290, 1–4.

[20] Zhang ZZ, Xue SX, Sun W, Zhang XT. Effects of stump sprouting regeneration and plant seedling afforestation of poplar I-69. Jiangsu Forestry Sci Technol. 1999; 4: 23–25.

[21] Shimaila R, Trevor CC, Bernard R, Glick. Isolation and characterization of new plant growth-promoting bacterial endophytes. Appl Soil Ecol. 2012; 61, 217–224.

[22] Asaf L Jonathan MC, Jeffery LD, Tanja W. Elucidating Bacterial Gene Functions in the Plant Microbiome. Cell Host Microbe. 2018; 24(4): 475–485. doi: 10.1016/j.chom.2018.09.005 30308154

[23] Ehrenfeld JG, Ravit B, Elgersma K. Feedback in the plant-soil system. Annu Rev Env Resour. 2005; 30(1): 75–115.

[24] Durand A, François M, Vanessa AL, Sarah G, Coralie B, Benoit V, et al. Bacterial diversity associated with poplar trees grown on a Hg-contaminated site: Community characterization and isolation of Hg-resistant plant growth-promoting bacteria. Sci Total Environ. 2018; 622–623: 1165–1177. doi: 10.1016/j.scitotenv.2017.12.069 29890585

[25] Knief C, Delmotte N, Chaffron S, Stark M, Innerebner G, Wassmann R, et al. Metaproteogenomic analysis of microbial communities in the phyllosphere and rhizosphere of rice. ISME J. 2012; 6, 1378–1390. doi: 10.1038/ismej.2011.192 22189496 PMC3379629

[26] Beckers B, Beeck OD, Michiel L, Weyens N, Boerjan W, Vangronsveld J. Structural variability and niche differentiation in the rhizosphere and endosphere bacterial microbiome of field-grown poplar trees. Microbiome. 2017; 5(1): 25–41. doi: 10.1186/s40168-017-0241-2 28231859 PMC5324219

[27] Hardoim PR, van Overbeek L.S, van Elsas JD. Properties of bacterial endophytes and their proposed role in plant growth. Trends Microbiol. 2008; 16, 463–471. doi: 10.1016/j.tim.2008.07.008 18789693

[28] Compant S, Clément C, Sessitsch A. Plant growth-promoting bacteria in the rhizo- and endosphere of plants: their role, colonization, mechanisms involved and prospects for utilization. Soil Biol Biochem. 2010; 42, 669–678.

[29] Jones JDG, Dangl JL. The plant immune system. Nature. 2006; 444, 323–329. doi: 10.1038/nature05286 17108957

[30] Yang R, Liu P, Ye W. Illumina-based analysis of endophytic bacterial diversity of tree peony (Paeonia Sect. Moutan) roots and leaves. Braz J Microbiol. 2017; 48, 695–705. doi: 10.1016/j.bjm.2017.02.009 28606427 PMC5628320

[31] Shakya M, Gottel N, Castro H, Yang ZK, Gunter L, Labbé J, et al. Amultifactor analysis of fungal and bacterial community structure in the root microbiome of mature *Populus deltoides* trees. PLoS One. 2013; 8, e76382. 24146861 10.1371/journal.pone.0076382 PMC3797799

[32] Gottel NR, Castro HF, Kerley M, Yang Z, Pelletier DA, Podar M, et al. Distinct microbial communities within the endosphere and rhizosphere of *Populus deltoids* roots across contrasting soil types distinct. Appl Environ Microb. 2011; 77, 5934–5944. 10.1128/AEM.05255-11 PMC3165402 21764952

[33] Bulgarelli D, Rott M, Schlaeppi K, Ver Loren van Themaat E, Ahmadinejad N, Assenza F, et al. Revealing structure and assembly cues for Arabidopsis root-inhabiting bacterial microbiota. Nature. 2012; 488, 91–95. doi: 10.1038/nature11336 22859207

[34] Lundberg DS, Lebeis SL, Paredes SH, Yourstone S, Gehring J, Malfatti S, et al. Defining the core Arabidopsis thaliana root microbiome. Nature. 2012; 488, 86–90. doi: 10.1038/nature11237 22859206 PMC4074413

[35] Edwards J, Johnson C, Santos-Medellín C, Lurie E, Podishetty NK, Bhatnagar S, et al. Structure, variation, and assembly of the root-associated microbiomes of rice. P Natl Acad Sci USA. 2015; 112, 911–920. 10.1073/pnas.1414592112 PMC4345613 25605935

[36] Liu GY, Chen LL, Shi XR, Yuan LY, Lock TR, Kallenbach RL. Changes in rhizosphere bacterial and fungal community composition with vegetation restoration in planted forests. Land Degrad Dev. 2019; 30(10): 1147–1157.

[37] Liang YM, Pan FJ, Ma JM, Yang ZQ, Yan PD. Long-term forest restoration influences succession patterns of soil bacterial communities. Environ Sci Pollut R. 2021; 28(16): 20598–20607. doi: 10.1007/s11356-020-11849-y 33405107

[38] Wang MH, Wan XH, Huang ZQ, Yu ZP, He ZM, Liu RQ. Changes of above- and belowground carbon input affected soil microbial biomass and community composition in two tree species plantations in subtropical China. Acta Ecol Sinica. 2016; 36, 3582–3590.

[39] Carson CM, Zeglin LH. Long-term fire management history affects N-fertilization sensitivity, but not seasonality, of grassland soil microbial communities. Soil Biol Biochem. 2018; 121, 231–239.

[40] Zhang Y, Cong J, Lu H, Li G, Qu Y, Su X, et al. Community structure and elevational diversity patterns of soil Acidobacteria. J Environ Sci. 2014; 26, 1717–1724. doi: 10.1016/j.jes.2014.06.012 25108728

[41] Fierer N, Bradford MA, Jackson RB. Toward an ecological classification of soil bacteria. Ecol. 2007; 88, 1354–1364. doi: 10.1890/05-1839 17601128

[42] Blumfield TJ, Xu ZH, Saffigna PG. Carbon and nitrogen dynamics under windrowed residues during the establishment phase of a second-rotation hoop pine plantation in subtropical Australia. Forest Ecol Manag. 2004; 200, 279–291.

[43] Krankina ON, Harmon ME, Griazkin AV. Nutrient stores and dynamics of woody detritus in a boreall forest: Modeling potential implications at the stand level. Can J Forest Res. 1999; 29, 20–32.

[44] Powers RF, Scott DA, Sanchez FG, Voldseth RA, Deborah PD, John D, et al. The North American long-term soil productivity experiment: Findings from the first decade of research. For Ecol Manag. 2005; 220: 31–50.

[45] Johnston-Monje D, Raizada MN. Conservation and diversity of seed associated endophytes in Zea across boundaries of evolution, ethnography and ecology. PLoS One. 2010; 6:e20396. 10.1371/journal.pone.0020396 PMC3108599 21673982

[46] Zhu WZ, Wang JX, Luo CR, Duan XM. Progresses of studies on forest sprout regeneration. Scientia Silvae Sinicae. 2007; 9: 74–82.

[47] Gould KA, Fredericksen TS, Morales F, Kennard D, Putz FE, Mostacedo B, et al. Post-fire tree regeneration in lowland Bolivia: implications for fire management. For Ecol Manage. 2002; 165, 225–234.

[48] Cruz A, Pérez B, Moreno JM. Resprouting of the Mediterranean-Type Shrub Erica australis with Modified Lignotuber Carbohydrate Content. J Ecol. 2003; 91: 348–356.

[49] Bill M, Chidamba L, Gokul JK, Labuschagne N, Korsten L. Bacterial community dynamics and functional profiling of soils from conventional and organic cropping systems. Appl Soil Ecol. 2021; 157: 103734.

[50] Philippe L, Manuel B, Daniel M, Moënne-Loccoz Y. Let the core microbiota be functional. Trends Plant Sci. 2017; 22(7): 583–595. doi: 10.1016/j.tplants.2017.04.008 28549621

[51] Hou P, Pan CD. Coarse woody debris and its function in forest ecosystem. Chinese J Appl Ecol. 2001; 2: 309–314. 11757388

[52] Garbelotto M. Root and butt rot diseases. Encyclop Forest Sci. 2004; 2: 750–758.

[53] Vries FTD, Griffiths RI, Knight CG, Nicolitch O, Williams A. Harnessing rhizosphere microbiomes for drought-resilient crop production. Science. 2020; 368: 270–274. doi: 10.1126/science.aaz5192 32299947

[54] Piri T. The spreading of the S type of *Heterobasidion annosum* from Norway spruce stumps to the subsequent tree stand. Eur J For Pathol. 1996; 26: 193–204.

[55] Walmsley JD, Godbold DL. Stump harvesting for bioenergy a review of the environmental impacts. Forestry. 2010; 83: 17–38.

[56] Cleary MR, Arhipova N, Morrison DJ, Thomsen IM, Sturrock RN, Vasaitis R, et al. Stump removal to control root disease in Canada and Scandinavia: A synthesis of results from long-term trials. For Ecol Manage 213; 290: 5–14.

[57] Hartmann A, Schmid M, van Tuinen D, Berg G. Plant-driven selection of microbes. Plant Soil. 2009; 321: 235–257.

[58] Andreote FD, Carneiro RT, Salles JF, Marcon J, Labate CA, Azevedo JL, et al. Culture-Independent Assessment of Rhizobiales-Related Alpha proteobacteria and the Diversity of Methylobacterium in the Rhizosphere and Rhizoplane of Transgenic Eucalyptus. Microb Ecol. 2009; 57 (1): 82–93. doi: 10.1007/s00248-008-9405-8 18536862

[59] Aira M, Gomez-Brandon M, Lazcano C, Baath EB, Domínguez J. Plant genotype strongly modifies the structure and growth of maize rhizosphere microbial communities. Soil Biol Biochem. 2010; 42: 2276–2281.

[60] Gaiero JR, McCall CA, Thompson KA, Day NJ, Best AS, Dunfield KE, et al. Ins the root microbiome: Bacterial root endophytes and plant growth promotion. AM J Bot. 2013; 100:1738–1750. 23935113 10.3732/ajb.1200572

[61] Donn S, Kirkegaard JA, Perera G, Richardson AE, Watt M. Evolution of bacterial communities in the wheat crop rhizosphere. Environ Microbiol. 2014; 17(3):610–621. doi: 10.1111/1462-2920.12452 24628845

